# Scrutinizing the triad of *Vibrio tapetis*, the skin barrier and pigmentation as determining factors in the development of skin ulcerations in wild common dab (*Limanda limanda*)

**DOI:** 10.1186/s13567-019-0659-6

**Published:** 2019-06-03

**Authors:** Maaike Vercauteren, Evelien De Swaef, Annelies M. Declercq, Hans Polet, Johan Aerts, Bart Ampe, Jesus L. Romalde, Freddy Haesebrouck, Lisa Devriese, Annemie Decostere, Koen Chiers

**Affiliations:** 1 Department of Pathology, Bacteriology and Avian Diseases, Faculty of Veterinary Medicine, Ghent University, Salisburylaan 133, 9820 Merelbeke, Belgium; 2 Department of Morphology, Faculty of Veterinary Medicine, Ghent University, Salisburylaan 133, 9820 Merelbeke, Belgium; 3 Research Division, Flanders Marine Institute (VLIZ), InnovOcean Site, Wandelaarkaai 7, 8400 Ostend, Belgium; 4 Research Institute for Agriculture, Fisheries and Food (ILVO), Animal Sciences Unit–Aquatic Environment and Quality, Ankerstraat 1, 8400 Ostend, Belgium; 5 Stress Physiology Research Group, Faculty of Sciences of Ghent University and Research Institute for Agriculture, Fisheries and Food (ILVO), Animal Sciences Unit, Green Bridge Science Park, 8400 Ostend, Belgium; 6 Research Institute for Agriculture, Fisheries and Food (ILVO), Animal Husbandry, Scheldeweg 68, 9090 Melle, Belgium; 7 Department of Microbiology and Parasitology, CIBUS–Faculty of Biology, Universidade de Santiago de Compostela, Campus Vida s/n, 15782 Santiago de Compostela, Spain

## Abstract

Recently, *Vibrio tapetis* was isolated for the first time from skin ulcerations in wild-caught common dab (*Limanda limanda*). To further examine its role in the development of these skin lesions, an in vivo experiment was performed. The significance of the skin barrier and in addition the difference between pigmented and non-pigmented side were investigated. Hence, the skin of common dab was treated in three different ways on both the pigmented and non-pigmented side. On a first “treatment zone”, the scales and overlying epidermal tissue were removed whereas in a second zone only the mucus was discarded. The third zone served as a non-treated zone. Thereafter, fish were challenged with *V. tapetis*. The control group was sham treated. Mortality, clinical signs, severity and size of the developing lesions were recorded. All animals were sacrificed and sampled 21 days post-inoculation. Significantly more fish of the group challenged with *V. tapetis* died compared to the control group with the highest incidence occurring 4 days post-inoculation. Fish challenged with *V. tapetis* developed more severe skin ulcerations. In zones where scales and epidermal tissue were removed, the ulcerations were more severe compared to zones where only mucus was eliminated. Ulcerations occurred more frequently, were more severe and larger on the pigmented side. Our data represents prove of *V. tapetis* as causative agent of ulcerative skin lesions although prior damage of the skin seems to be a major contributing factor. Furthermore, the pigmented side seemed predisposed to the development of skin ulcerations.

**Electronic supplementary material:**

The online version of this article (10.1186/s13567-019-0659-6) contains supplementary material, which is available to authorized users.

## Introduction

*Vibrio tapetis* is a well-known pathogen causing brown ring disease in various clam species such as the manila clam (*Ruditapes philippinarum*) and carpet-shell clam (*R. decussatus*) [1, 2]. Only a handful of studies describe the isolation of *V. tapetis* from skin lesions and internal organs of moribund cultivated or captive held aquatic vertebrates, namely corkwing wrasse (*Symphodus melops*) [3], Atlantic halibut (*Hippoglossus hippoglossus*) [4], wedge sole (*Dicologlossa cuneata*) [5], Dover sole (*Solea solea*) [6] and two native fish species reared in Chile (*Genypterus chilensis* and *Paralichthys adspersus*) [7]. Recently, *V. tapetis* was isolated for the first time from skin ulcerations, lesions affecting epidermal and dermal tissues, in wild-caught common dab (*Limanda limanda*) in the Belgian part of the North Sea (BNS) [8].

The wild common dab populations in this geographic area showed a recent prevalence of skin ulcerations up to 3.5% [9]. Various possible causal agents, ranging from infectious agents to biological toxins, were suggested as involving factors in the development of skin ulcerations [10–12]. Nevertheless, the etiology of these lesions is largely unknown and many questions remain regarding their development and possible contributing factors in terms of fish health and environmental conditions. Besides being a welfare issue, skin ulcerations in wild fish may also pose limitations to the productivity of the marine ecosystem [11, 13] and diminish the economic value of the fish [11], urging for an understanding of the cause(s) of these lesions.

All marine organisms, including fish, live in close contact with their environment, containing potential pathogenic micro-organisms [14]. Hence, the skin acts as an important barrier between the fish and its environment. The outermost part of the skin consists of the epidermal layer and the covering mucus layer; two complex structures offering mechanical, chemical and immunological protection against pathogenic agents [15, 16]. Changes in one or both layers, caused by abrasion, stress or pollutants, may increase the susceptibility of the fish for adherence and subsequent invasion of microorganisms and hence for the development of skin ulcerations, as demonstrated for *Tenacibaculum maritimum* in four marine species [17].

Common dab and flatfish in general, have a fiercely discussed asymmetric morphology, with the pigmented (P) side facing the water column and the non-pigmented (NP) side being in closer interaction with the sediment [18]. Based on these differences, a divergent morphological composition and possibly linked varying susceptibility to the development of skin ulcerations is plausible. A handful of studies on this issue are available but inconsistent ranging from a clear to a sex-related difference regarding the development of skin ulcerations on both sides [19–22].

In view of this, the aim of the present study was to (i) pinpoint the ability of *V. tapetis* to induce skin ulcerations in common dab; (ii) assess the impact of prior skin damage on the development of skin ulcerations and (iii) determine differences between the pigmented and non-pigmented side on the development of skin ulcerations.

## Materials and methods

### Animals and housing facilities

Sixty common dab were caught during short fishing hauls (± 10 min) using a 6 m beam trawl on board of the research vessel (RV) “Simon Stevin” at two sampling locations [L1 (N51°10.344; E2°38.699) and L2 (N51°9.886; E2°34.797)] situated in the western segment of the BNS, within three miles offshore. On board of the RV, only fish in good condition and with a minimal length of 17.5 cm were collected and immediately placed in a survival tank (1 × 1.2 × 1 m; 640 L) with continuous water renewal. Within 4 h after capture, all fish were transferred to circular tanks (diameter 2.6 m, 4000 L; 30 fish per tank) with sand-covered bottoms (6 cm layer thickness, 0–2 mm grain size) and filled with recirculating, filtered natural seawater at the Marine Station Ostend (Flanders Marine Institute, VLIZ, Ostend, Belgium). The water quality was monitored daily and kept in pre-set ranges (16.3 ± 0.4 °C; pH 8.1 ± 0.0; 33.8 ± 0.2 PSU; 85.3 ± 1.1% oxygen saturation). Ammonia and nitrite levels never exceeded 0.1 and 1 mg L^−1^, respectively. Fish were fed three times a week with chopped whiting (*Merlangius merlangus*) (10 g per fish). After an acclimatization period of 18 days, the fish were transported to the experimental units using small transportation boxes (39.4 × 59.8 × 18.6 cm; 30 L) supplied with an oxygen tablet (JBL GmbH & Co.KG, Germany).

Upon arrival at the experimental units, fish were randomly divided over five tanks (1 × 1 × 0.5 m; 450 L) with each tank containing 12 fish. The tanks were filled with recirculating and aerated seawater, half natural and half artificial (Instant ocean, Aquarium system, France) and a sand layer (6 cm layer thickness, 0–2 mm grain size). Water parameters were monitored daily. Throughout the experiment, the following averages were recorded: 15.3 ± 1.0 °C, pH 8.4 ± 0.1; 32.5 ± 1.5 PSU and 79.5 ± 6.6% oxygen saturation. Ammonia and nitrite levels never exceeded 0.2 and 1 mg L^−1^, respectively. Upon water renewal, artificial seawater was added. Fish were fed every 2 days with chopped Pangas catfish (*Pangasius pangasius*) (10 g per fish). Feeding time lasted for 1 h after which the remaining feed was removed. One week after arrival, all fish were deprived of food 24 h prior to the experimental trial. The experiment was approved by the Ethical Committee of the Faculty of Veterinary Medicine and Bio-engineering Sciences, Ghent University (EC 2015_89).

### Bacterial isolate

The *V. tapetis* isolate was recovered in pure culture from an ulcerative skin lesion of a common dab caught in the BNS [8].

For the experimental challenge, the *V. tapetis* isolate was cultured on tryptic soy agar (TSA, Sigma Aldrich N.V., Belgium) supplemented with 1.5% NaCl and incubated at 16 ± 1 °C for 3 days. Colonies were subsequently transferred to 4 mL tryptic soy broth (TSB, Sigma Aldrich) supplemented with 1.5% NaCl. After a 24 h incubation period at 16 ± 1 °C, the broth was used to inoculate 36 mL TSB medium supplemented with 1.5% NaCl. These cultures were again incubated for 16 h (16 ± 1 °C), after which the cultivated broth was centrifuged (2465 × *g*, 2 × 10 min, 16 °C), and the pellet re-suspended in 40 mL autoclaved artificial seawater. Bacterial titers were verified by making an eightfold dilution series in triplicate on TSA plates supplemented with 1.5% NaCl, prior to administration.

### Experimental design

Following anesthesia using tricaine methanosulfonate (MS-222; 100 mg L^−1^; Sigma Aldrich), a tag (T-bar anchor tag, Floy Tag Inc., USA) was inserted in the caudal epaxial musculature of all fish. The weight of the fish (W_B_) was registered.

On both the P and NP side, three distinct square “treatment zones” (± 2.3 cm^2^) were defined, with an interspace of 0.5 cm (Additional file 1). In one zone, the scales and overlying epidermis were removed by scraping with a scalpel (mechanical treatment, MT). In a second zone, the mucus layer on top of the epidermis was removed using 80% ethanol (chemical treatment, CT) [23, 24]. The third zone was the non-treatment zone (NT) where the skin was left intact and therefore served as the control zone. The sequence of all treatments was altered on each fish as follows: (subgroup 1) MT–CT–NT; (2) MT–NT–CT; (3) NT–MT–CT; (4) NT–CT–MT; (5) CT–NT–MT; (6) CT–MT–NT, resulting in six subgroups (1–6) of 10 fish each (Additional file 1).

Following recovery, all fish were divided into two groups; fish which were to be challenged with *V. tapetis* and a sham-treated control group, including three and two tank replicates (henceforth referred to as replicates), respectively. Each replicate contained two fish of each subgroup (1–6: see above) hence a total of 12 fish which were placed in a separate tank containing 35 L artificial seawater (55 × 38 × 28 cm) (Additional file 1). The bacterial suspension of *V. tapetis* was added to the water of the three tank replicates resulting in a final concentration of 3.3 × 10^5^ colony forming units (CFU) mL^−1^ (challenge group, *n* = 36). The fish of the two tank replicates constituting the control group (*n* = 24) were subjected to the same procedures as the challenge group, but without addition of the bacterial suspension. After 60 min, the fish of each replicate were transferred to the 450 L tank after which clinical signs and mortality were recorded on a daily basis during 21 days post-inoculation (dpi). Every second day, starting 5 dpi, all fish were netted, clinically inspected and the P and NP side of each fish photographed to determine the “gross ulceration score” (GUS), “total affected area” (TA) and “ulcerative area” (UA) (see further). As soon as the humane endpoints (extensive lesions and/or subcutaneous hemorrhages, weak or no reaction upon handling) were observed, the fish was euthanized using an overdose of MS-222 (500 mg L^−1^) and necropsied as described below. At 21 dpi, all surviving fish were sacrificed by an overdose of MS-222 and necropsied as outlined further.

### Post-mortem examination

All fish were measured (standard length, L) and weighed (W_E_) to calculate the Fulton condition factor [25] (K_E_ = 100 * (W_E_/L^3^)) enabling a comparison with the body condition of the fish before the treatment (K_B_ = 100 * (W_B_/L^3^)). The gender of the fish was recorded and both sagittal otoliths were collected to determine the age using the method described by Imsland et al. [26]. A parasitological examination was performed using wet mount preparations of gill biopsies and skin mucus samples. Fish were photographed on both sides to evaluate the presence and/or type of lesions and to calculate the GUS, TA and UA (see further). Finally, a full necropsy was performed whereby samples of the skin and internal organs were collected for bacteriological and histological examination.

### Skin lesion assessment

#### Gross ulceration score (GUS)

To quantify the severity of an ulcerative skin lesion that developed in the treatment zones (MT–CT–NT), a scoring system was applied. The “gross ulceration score” (GUS) was defined as the sum of the scores of seven parameters relating to the depth, healing, elevation of the edge of the lesion, pigmentation in or around the lesion, color of the lesion, hemorrhages around the lesion and the shape of the lesion (Additional file 2). Inherently, ulcerations with a higher GUS reflect a more severe lesion. Scoring was conducted blindly using photographs of all fish collected every second day, starting at 5 dpi and on the day the fish died.

#### Total affected area (TA) and ulcerative area (UA)

To quantify the extent of the ulceration, the area of the fish’s skin affected by the lesion was determined. The “Total affected area” (TA) of each treatment zone (MT–CT–NT) was calculated using scientific image analysis software (ImageJ 1.4) and represents the total skin area affected by the ulceration, including the healing edge and/or hemorrhages around the lesion. The surface of the open, active lesion is named the “Ulcerative area” (UA).

### Bacteriological examination

Skin samples of all treatment zones (MT–CT–NT), as well as samples of liver, kidney and spleen, were inoculated on thiosulfate citrate bile salt sucrose agar (TCBS; Sigma Aldrich) and incubated for 3 days at 16 ± 1 °C. *V. tapetis* was identified by a PCR-based method using REP1D and REP2D sequences [27].

### Histological examination

Tissue samples of all skin treatment zones (MT–CT–NT), as well as samples of gill, liver, spleen, intestine, kidney and heart were fixated for 24 h in a phosphate-buffered 4% formaldehyde solution. Tissues were processed according to standard techniques, sectioned (5 µm) and stained with hematoxylin and eosin (H&E).

### Immunohistochemistry

Demonstration of *V. tapetis* in a subset of skin samples (at least 20% of the fish per group) by means of immunohistochemistry was performed as described previously [8]. In short, 5 µm tissue sections were incubated with antibodies against *V. tapetis* (1:500). Subsequently, bound antibodies were visualized using the DAKO Envision + System/HRP diaminobenzidine (DAB+) staining kit (DakoCytomation, Glostrüp, Denmark).

### Data processing and statistical analysis

To investigate the difference in mortality between the control and challenge group, a logistical regression was used. Host-specific data namely length, weight, body condition, gender and age gathered during clinical examination was analyzed using a linear mixed model.

To study the role of *V. tapetis* and the effect of the three treatments in the development of skin ulcerations, the total experimental follow up was divided into three distinct periods: 0–5 dpi, 6–15 dpi and 16–21 dpi. For each period, a distinction was made between fish that died before and fish that survived until the end of the experimental period (21 dpi). The fish that died before the end of the experimental period were excluded from the statistical analysis for estimating the differences in GUS, TA and UA. Thus, only the surviving fish were included in the analyses on 5, 15 and 21 dpi. The resulting data of fish that died during the experiment were reported in a descriptive manner. For estimating the difference in GUS, TA and UA, a linear mixed model (proc GLIMMIX) was applied, followed by pairwise comparisons using a Tukey–Kramer adjustment for multiple testing. GUS, TA and UA were used as response variables. Different treatments (MT–CT–NT), groups, replicates, and subgroups (variable order of the treatments) and side (P or NP) were implemented as co-variables. Analysis on differences between P and NP side was performed in the same manner with side and group as response variables. Interaction effects were studied to estimate group-dependency. In all models, tank was included as a random intercept.

Statistical results were considered to be significant when p-values were lower than 0.05. A *p*-value between 0.05 and 0.1 was considered as a trend. All statistical analyses were performed using SAS 9.4 and graphs were constructed using R Studio.

## Results

The main fish characteristics are listed in Table 1. The mean length of the fish was 21.7 ± 2.8 cm in the challenge and 22.8 ± 3.0 cm in the control group. In total, 21 males and 39 females were included with 16 males and 20 females and 5 males and 19 females in the challenge and the control group, respectively. The mean age of fish included in the challenge group (35.4 ± 12.6 months) was slightly lower, albeit not significantly, compared to the age of the control group (37.4 ± 12.0 months) (*p* = 0.6703). The fish from the challenge (K_B_ = 0.99 ± 0.1) and control (K_B_ = 1.0 ± 0.2) group displayed a similar body condition at the beginning of the study (K_B_; *p* = 0.3958) remaining stable throughout the total experimental period (K_B_ − K_E_) with no difference between challenged and control fish (*p* = 0.6144).

**Table 1:** **Main fish characteristics (length, weight, body condition, gender and age) per tank replicate and per group as well as mortality**

Group	Replicate	No.	Length (cm)	Weight (g)		Body condition		Gender	Age (months)	Mortality		
				W_B_	W_E_	K_B_	K_E_			< 5 dpi	< 15 dpi	< 21 dpi
Challenge	1	12	22.6 ± 3.4	119.8 ± 51.7	116.5 ± 50.2	0.99 ± 0.09	0.96 ± 0.1	5 M/7 F	41.0 ± 14.8	2	1	0
	2	12	20.6 ± 2.2	86.6 ± 26.6	85.9 ± 30.4	0.96 ± 0.09	0.9 ± 0.09	6 M/6 F	29.7 ± 5.6	6	2	0
	3	12	21.7 ± 2.8	105.7 ± 39.3	106.3 ± 39.3	1.0 ± 0.08	0.99 ± 0.1	5 M/7 F	35.0 ± 13.4	8	1	0
	Mean		21.7 ± 2.9	104.0 ± 41.7	103.8 ± 42.1	0.99 ± 0.1	0.96 ± 0.09	16 M/20 F	35.4 ± 12.6			
Control	1	12	22.8 ± 2.9	124.3 ± 56.4	120.1 ± 54.6	1.0 ± 0.2	0.97 ± 0.08	2 M/10 F	38.5 ± 7.8	0	0	1
	2	12	22.9 ± 3.2	130.2 ± 58.3	128.8 ± 61.1	1.0 ± 0.1	0.99 ± 0.09	3 M/9 F	36.3 ± 15.5	2	0	1
	Mean		22.8 ± 3.0	127.3 ± 56.2	124.3 ± 56.7	1.0 ± 0.2	0.98 ± 0.08	5 M/19 F	37.4 ± 12.0			

W_B_: weight at the beginning of the experimental period, W_E_: weight at the end of the experimental period, K_B_: Fulton condition index at the beginning of the experimental period, K_E_: Fulton condition index at the end of the experimental period, M: male fish, F: female fish.

In one fish of the control group, a parasite resembling *Lernanthropus* spp. was noted on the gills. In two fish of the challenge group, a parasite with a morphology similar to *Tetrahymena* spp. was found on the skin mucus albeit in low numbers.

Length (*p* = 0.2190), weight (W_B_: *p* = 0.1635; W_E_: *p* = 0.2191), body condition (K_B_ − K_E_: *p* = 0.6144) and age (*p* = 0.6703) of fish included in the control group showed no significant differences with fish from the challenge group and were therefore not implemented in further analyses. Furthermore, gender and parasitic load were neither implemented in further analyses.

A significantly higher mortality was found in the challenge group (55.6%) compared to the control group (16.7%) (*p* = 0.0060). The main mortality peak was encountered on 4 dpi whereby 12 out of 36 challenged fish died (Additional file 3).

No macroscopic abnormalities were seen in the gills and internal organs from the fish of the challenge or the control group upon necropsy. These findings were confirmed by histological examination.

### Chronological changes in skin lesions following challenge with *V. tapetis*

#### Non-treated (NT-) zone

No ulcerations developed neither in the challenge nor in the control group. *V. tapetis* was isolated from the NT-zone in two challenged and two control fish at 21 dpi.

#### Chemical treatment (CT-) zone

In the control group, nine out of the 24 fish developed skin ulcerations in the CT-zone. One of these fish died at 4 dpi with a severe ulceration with a GUS of 9, TA of 3.1 cm^2^ and UA of 3.2 cm^2^. In two out of eight surviving fish, the ulceration had healed at 21 dpi, the other six fish showed signs of healing. In the challenge group, 16 out of the 36 fish developed skin ulcerations in the CT-zone. Nine and three fish died before 5 and 15 dpi, respectively. The average GUS, TA and UA scores of the CT-zone ulcerations are listed in Table 2.

**Table 2:** **Mean gross ulceration score (GUS), total affected area (TA, cm** ^**2**^ **) and ulcerative area (UA, cm** ^**2**^ **) of the ulcerations in the chemically treatment zones in challenged and control fish**

Group	Dead or surviving	0–5 dpi			6–15 dpi			16–21 dpi		
		GUS	TA	UA	GUS	TA	UA	GUS	TA	UA
Challenge	D	4.5 ± 5.2	1.6 ± 2.9	1.6 ± 2.9	NA	NA	NA	NA	NA	NA
Control	D	7.8 ± 2.6	1.5 ± 0.9	1.1 ± 1.2	6.7 ± 0.6	1.7 ± 0.5	1.3 ± 1.1	NA	NA	NA
Challenge	S	7.0 ± 1.0	0.8 ± 0.4	0.6 ± 0.6	7.8 ± 4.9	0.7 ± 0.6	0.4 ± 0.7	5.6 ± 1.1	0.4 ± 0.3	0.1 ± 0.2
Control	S	8.4 ± 5.0	0.9 ± 0.6	0.7 ± 0.7	6.5 ± 2.5	0.3 ± 0.2	0.2 ± 0.1	4.3 ± 2.5	0.3 ± 0.2	0.1 ± 0.3

Values are subdivided according to period with 0–5, 6–15 and 16–21 days post-inoculation (dpi). A subdivision was made between fish that died during the experimental period (D) and fish that survived in that period (S).

NA: not available.

The difference between the GUS, TA and UA of the ulcerations in the CT-zone of surviving fish were not statistically significant between the control and challenged fish throughout the entire experimental period, based on the analyses at 5 dpi (GUS: *p* = 0.0729; TA: *p* = 0.6443; UA: *p* = 0.6933), 15 dpi (GUS: *p* = 0.7525; TA: *p* = 0.7976; UA: *p* = 0.9136) and 21 dpi (GUS: *p* = 0.9968; TA: *p* = 1.0000; UA: *p* = 0.9814).

### Mechanical treatment (MT-) zone

The GUS, TA and UA scores of MT-zone ulcerations are listed in Table 3.

**Table 3:** **Mean gross ulceration score (GUS), total affected area (TA) and ulcerative area (UA) of the ulcerations in the mechanically treatment zones in challenged and control fish**

Group	Dead or surviving	0–5 dpi			6–15 dpi			16–21 dpi		
		GUS	TA (cm^2^)	UA (cm^2^)	GUS	TA (cm^2^)	UA (cm^2^)	GUS	TA (cm^2^)	UA (cm^2^)
Challenge	D	9.3 ± 2.3	2.1 ± 1.2	1.5 ± 1.3	8.0 ± 3.6	1.7 ± 0.9	1.0 ± 0.8	NA	NA	NA
Control	D	7.5 ± 2.1	2.6 ± 2.4	1.8 ± 2.8	NA	NA	NA	3.8 ± 3.3	0.6 ± 0.6	0.1 ± 0.1
Challenge	S	8.5 ± 2.3	1.5 ± 0.9	1.1 ± 0.9	7.0 ± 2.6	1.2 ± 0.9	0.4 ± 0.6	6.0 ± 2.8	1.1 ± 0.7	0.3 ± 0.5
Control	S	5.4 ± 4.0	1.1 ± 1.3	0.2 ± 0.3	4.4 ± 3.5	0.8 ± 0.8	0.1 ± 0.2	4.5 ± 2.8	0.7 ± 0.7	0.1 ± 0.2

Values are subdivided according to period with 0–5, 6–15 and 16–21 days post-inoculation (dpi). A subdivision was made between fish that died during the experimental period (D) and fish that survived in that period (S).

NA: not available.

#### 0–5 dpi

Two fish of the control group died at 4 dpi. One fish showed small ulcerations with a GUS of 6.5 ± 2.1 and TA of 0.9 ± 0.7 cm^2^. The other fish was euthanized as it showed extensive hemorrhages and large and severe ulcerations. These ulcerations had a GUS of 8.5 ± 2.1 and TA of 4.3 ± 2.3 cm^2^. *V. tapetis* was not isolated from any fish. Immunohistochemical analysis of a skin ulceration of the second fish on the P side revealed small numbers of comma-shaped immunopositive cells at the edge of the ulceration on top of the stratum spongiosum (dermis) not invading the muscular tissue.

Merely all fish of the control group (*n* = 22) that were alive at the end of the first experimental period showed ulcerations with a GUS of 5.4 ± 4.0. The TA of the ulcerations was 1.1 ± 1.3 cm^2^ whereby 0.2 ± 0.3 cm^2^ was observed to be active (UA).

**Figure 1:**
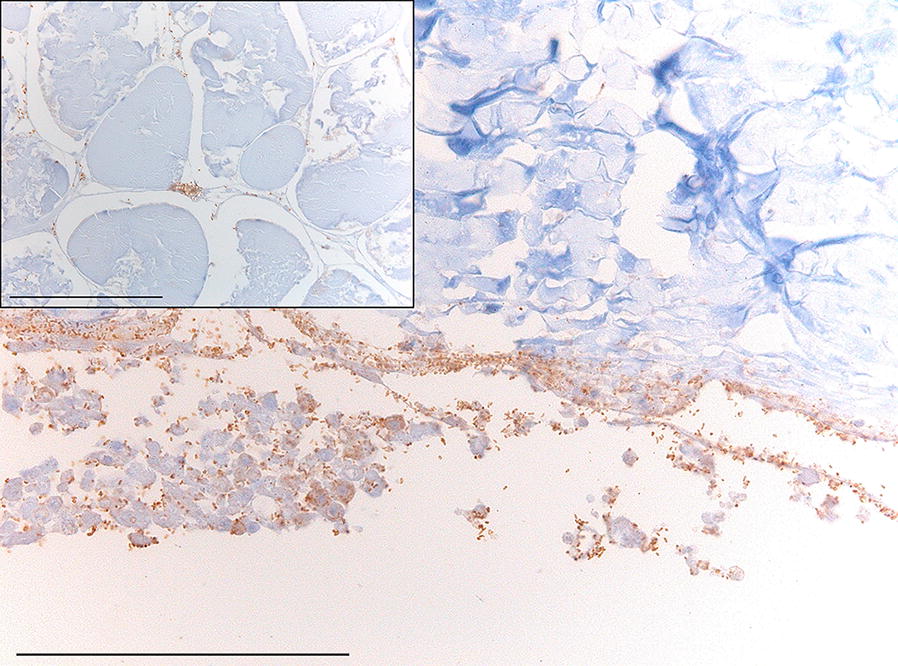
**Immunohistochemical results demonstrating the presence and location of comma-shaped immunopositive cells (brown).** Example of a skin ulceration of a challenged fish that died at 4 days post-inoculation. In the exposed dermal tissue and interstitial space of the underlying muscular tissue comma-shaped immunopositive cells are noted. The insert shows a higher magnification to point out the invasion of the immunopositive cells in the interstitial spaces of the muscle. Scale bar = 100 µm.

In the challenge group, 16 fish died before 5 dpi, mainly on 4 dpi (Table 1). The observed skin ulcerations in these fish had a GUS of 9.3 ± 2.3, with a TA and UA of 2.1 ± 1.2 cm^2^ and 1.5 ± 1.3 cm^2^, respectively. From three of five sampled fish, *V. tapetis* was isolated from the ulceration. One of the fish that died at 5 dpi harbored a pure culture of *V. tapetis* in the spleen. Immunohistochemical analyses of the ulcerations of eight sampled fish revealed the presence of comma-shaped immunopositive cells. The cells were located at the ulcerated surface and in underlying dermal tissue. In all cases, the immunopositive cells infiltrated into the muscular tissue superficially, with occasional observations of the cells in the muscular connective tissue (Figure 1).

At 5 dpi, the remaining individuals of the challenge group (*n* = 20) all showed ulcerations with a GUS of 8.5 ± 2.3. The ulcerations had a TA of 1.5 ± 0.9 cm^2^ and UA of 1.1 ± 0.9 cm^2^.

**Figure 2:**
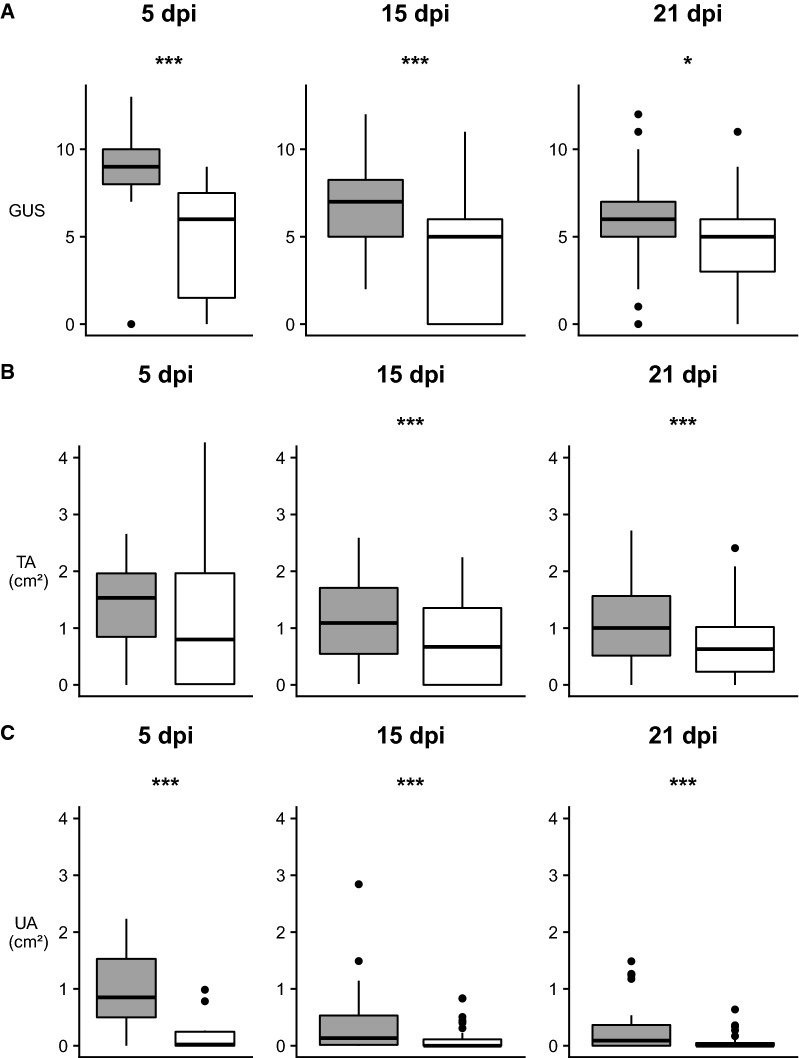
**Overview of skin lesion assessment for mechanically treated zones during the experimental period.** Comparison of the main parameters between challenge (grey) and control (white) groups at 5, 15 and 21 days post-inoculation (dpi). Only the fish that were alive at the end of the experimental period (21 dpi) are depicted. Three asterisks indicate a significant difference between challenge and control group, one asterisk represents a trend. **A** Gross ulcerations score (GUS) were significantly higher in challenge group at 5 and 15 dpi. At 21 dpi the same trend was visible. **B** Total affected area (TA) was higher in challenge group compared to the control group at 15 and 21 dpi. **C** Ulcerative area (UA) was higher in challenge group compared to the control group during the entire experimental period.

The GUS (*p* = 0.0035) and UA (*p* < 0.0001) of the surviving fish at 5 dpi were significantly higher in the challenge group compared to the control group (Figures 2A and C). The TA did not show a significant difference between challenge and control groups (*p* = 0.6682) (Figure 2B).

#### 6–15 dpi

None of the control fish died. The GUS at 15 dpi was 4.4 ± 3.5. TA and UA of the ulcerations 0.8 ± 0.8 cm^2^ and 0.1 ± 0.2 cm^2^, respectively.

In the challenge group, four out of 20 remaining fish died between 6 and 15 dpi. Ulcerations were found in all fish (GUS 8.0 ± 3.5). The TA and UA were 1.7 ± 0.9 cm^2^ and 1.0 ± 0.8 cm^2^, respectively. In two of the four fish, *V. tapetis* was isolated from the liver. *V. tapetis* was not retrieved from the ulcerations on the MT-zone. In two of the four fish, comma-shaped immunopositive cells were visualized in the skin ulceration using immunohistochemical staining.

The remaining challenged fish (*n* = 16) all showed ulcerations with GUS, TA and UA values of 7.0 ± 2.6, 1.2 ± 0.9 cm^2^ and 0.4 ± 0.6 cm^2^, respectively.

At 15 dpi, the GUS of the ulcerations of the challenge group was significantly higher compared to the control group (*p* < 0.0001) (Figure 2A). A statistically significant difference was noted for the TA and UA between the challenge and control group (*p* < 0.0001 and *p* = 0.0002, respectively) (Figures 2B and C).

#### 16–21 dpi

Between 16 and 21 dpi, two control fish died. The ulcerations in the MT-zone had a GUS of 3.8 ± 3.3, TA of 0.6 ± 0.6 cm^2^ and UA of 0.1 ± 0.1 cm^2^. *V. tapetis* was not isolated and immunohistochemical analysis did not reveal immunopositive cells in the ulcerations.

The remaining control fish (*n* = 20) were sacrificed at 21 dpi, of which 14 and six fish had ulcerations on both sides or only on the P side, respectively. The ulcerations had a GUS of 4.5 ± 2.8. The TA was 0.7 ± 0.7 cm^2^ whereby 0.1 ± 0.2 cm^2^ was observed to be active (UA). From one fish, *V. tapetis* was isolated from an ulceration in the MT-zone. This was not confirmed by immunohistochemical staining.

None of the 16 remaining challenged fish died. At 21 dpi, the ulcerations found in the sacrificed fish had a GUS, TA and UA of 6.0 ± 2.8, 1.1 ± 0.7 cm^2^ and 0.3 ± 0.5 cm^2^, respectively. *V. tapetis* was isolated from the ulcerations of four fish. The immunohistochemical analysis did not reveal immunopositive cells in the ulcerations of any of the sampled fish.

At 21 dpi, a trend of a higher GUS of ulcerations of challenged compared to control fish (*p* = 0.0600) was noted (Figure 2A). The TA (*p* = 0.0030) and UA (*p* = 0.0006) remained significantly higher in the MT-zone ulcerations of the challenge group compared to the control group (Figures 2B and C).

The average GUS, TA and UA scores of the ulcerations in the MT-zones were significantly higher compared to the CT-zones at 5 dpi (GUS: *p* = 0.0058; TA: *p* = 0.0097; UA: *p* = 0.0353), 15 dpi (GUS: p = 0.0035; TA: *p* = 0.0029; UA: *p* = 0.0410) and 21 dpi (GUS: *p* = 0.0026; TA: *p* = 0.0028; UA: *p* = 0.0333). These results were independent from the group (control or challenge).

Histological examination confirmed the gross appearance of the lesions. Ulcerations were characterized by focal loss of epidermal and/or dermal tissue. The dermis was often infiltrated by mild to moderate amounts of inflammatory cells. Underlying muscle tissue was regularly degenerated and/or infiltrated by inflammatory cells. Partially healed ulcerations were mainly typified by a one or two cell-layered epidermis overlying a disrupted dermal tissue without the presence of scales and/or scale pockets. Inflammatory cells were present in moderate amounts and were localized mainly in dermal and muscular tissue. Hemorrhages were rather moderate. These observations were similar in the challenge and the control group and in lesions occurring on the P and NP side.

### Chronological changes in skin lesions on the pigmented and non-pigmented side

Since the main difference between the challenge and control group in the development of skin ulcerations was found in the MT-zones, only these zones are discussed when comparing the ulcerations in the pigmented (P) and non-pigmented (NP) sides of the fish.

At 5, 15 and 21 dpi, the difference between both sides was not dependent on the group the fish belonged to (control or challenge). Therefore, in the statistical analysis, the results of the control and challenged fish were taken together.

#### 0–5 dpi

**Figure 3:**
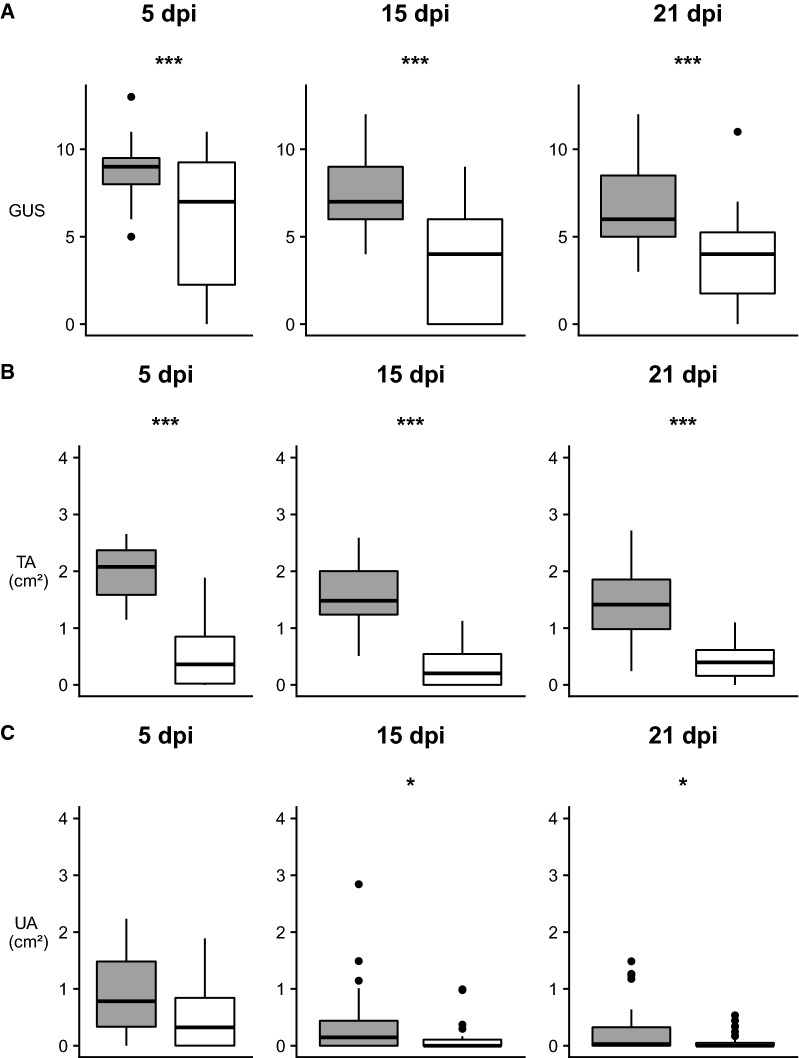
**Overview of skin lesion assessment for mechanically treated zone ulcerations on pigmented and non-pigmented sides.** Comparison of the main parameters between pigmented (grey) and non-pigmented (white) sides at 5, 15 and 21 days post-inoculation (dpi). Results of the control and challenge fish were taken together. Only the fish that were alive at the end of the experimental period (21 dpi) are depicted here. Three asterisks indicate a significant difference between pigmented and non-pigmented side, one asterisk represents a trend. **A** Gross ulcerations score (GUS) and **B** total affected area (TA) were higher on the pigmented side compared to the non-pigmented side during the entire experimental period. **C** Ulcerative area (UA) did not differ significantly although a similar trend was visible at 15 and 21 dpi.

Merely all surviving fish (*n* = 42) developed ulcerations on both P and NP sides except for four fish with only an ulceration on the P side. The GUS of the ulcerations developed on P and NP side at 5 dpi was 8.6 ± 1.8 and 6.1 ± 4.1, respectively. TA and UA of ulcerations were 2.2 ± 0.9 cm^2^ and 1.0 ± 1.1 cm^2^ on the P side and 0.6 ± 0.6 cm^2^ and 0.5 ± 0.6 cm^2^ on the NP side, respectively. Generally, the ulcerations on the P side were significantly more severe (GUS, *p* = 0.0321) and larger (TA, *p* = 0.0066) compared to the NP side (Figures 3A and B). The part of the ulcerations that was active (UA) was similar between the P and NP side (*p* = 0.1580) (Figure 3C).

#### 6–15 dpi

At 15 dpi, all fish (*n* = 38) had developed an ulceration on the P side while only 24 fish displayed an ulceration on the NP side. The GUS of the ulceration on the P side was significantly higher (7.5 ± 2.3) compared to the GUS of the ulcerations on the NP side (3.5 ± 3.1) (*p* = 0.0058) (Figure 3A). The TA of the ulcerations was 1.6 ± 0.7 cm^2^ on the P side whereby 0.4 ± 0.6 cm^2^ was found to be active (UA). On the NP side, TA of the ulcerations was 0.3 ± 0.4 cm^2^ with UA of 0.1 ± 0.2 cm^2^. The TA was significantly higher in P side compared to the NP side (*p* = 0.0017) and the same trend was present upon considering the UA (*p* = 0.0538) (Figures 3B and C).

#### 16–21 dpi

Of the surviving fish (*n* = 36), 29 had developed an ulceration on both sides and 7 fish showed ulcerations only on the P side. The GUS of the ulcerations on the NP side was 3.6 ± 2.6. The TA and UA of the latter were 0.4 ± 0.3 cm^2^ and 0.1 ± 0.1 cm^2^, respectively. The ulcerations on the P side had a GUS of 6.7 ± 2.1, TA of 1.4 ± 0.6 cm^2^ and UA of 0.3 ± 0.5 cm^2^. Comparing ulcerations on the P side and NP side, at 21 dpi, ulcerations on the P side were generally more severe (*p* = 0.0126) and had a larger surface (TA; *p* = 0.0029) compared to the ulcerations on the NP side (Figure 3). The UA showed a similar trend (*p* = 0.0528).

## Discussion

The results of the present study strongly point towards the involvement of *V. tapetis* in skin ulcerations since more common dab inoculated with a bacterial suspension of *V. tapetis* (3.3 × 10^5^ CFU mL^−1^, an inoculation dose comparable to another study [28]) developed skin ulcerations compared to control fish that were sham-treated. Furthermore, skin ulcerations were significantly more severe and covered a larger surface in the challenged fish. Following bacteriological analysis, *V. tapetis* was isolated from skin ulcerations of eight fish in total and using immunohistochemical staining, comma-shaped immunopositive cells, presumed to be *V. tapetis* were visualized in these skin ulcerations. Notably, *V. tapetis* was isolated predominantly from the ulcerations of fish that died at 4 dpi. This observation was again supported by immunohistochemical staining where mainly in those fish the comma-shaped immunopositive cells were visualized.

To determine the role of the skin or mucus as a protective barrier against infection and colonization by *V. tapetis*, three treatments were applied on the skin. After inoculation with *V. tapetis*, ulcerations developed predominantly in the mechanically treated zone where scales and overlying epidermis were removed. Moreover, the ulcerations in this zone were more severe compared to ulcerations in chemically treated zone where the mucus was locally removed using ethanol. In the non-treated zone that served as control zones, no skin ulcerations developed.

The mechanical treatment removes the scales and overlying epidermis which leaves the dermal tissue exposed hereby resembling wounds inflicted by predators or fishing gear at sea [29]. Exposure of dermal tissue could facilitate the adherence of *V. tapetis* to, e.g. exposed fibronectin and collagen. The virulence factor(s) of *V. tapetis* that could possibly be involved in this process are not yet elucidated, although adhesion components such as pili were described in strains pathogenic to mollusks [1, 30]. The adhesion may be followed by the further destruction and invasion of the host tissue. Based on immunohistochemical staining in the present study, it was shown that comma-shaped immunopositive cells, presumed to be *V. tapetis,* were able to invade the dermis and adjacent muscular tissue, which is often mediated by several enzymes degrading the extracellular matrix. In *V. tapetis* isolates pathogenic to mollusks, 87 different proteins including phospholipases and proteases were demonstrated in their secretome [30]. However, at present, no such data are available for *V. tapetis* isolates collected from fish.

The chemical treatment removed the mucus layer, which has various protective properties, both mechanical and immunochemical [15, 16]. The mucus contains various antibacterial proteins such as paradaxin, pleurocidin and parasin 1 and quickly accumulates in a wound region forming a protective layer [31, 32]. The results of the present study suggest that *V. tapetis* is to a lesser extent hampered by the mucus layer since less fish developed skin ulcerations in the chemical treatment zones. However, it is difficult to make a clear statement since, according to the “dynamic mucus coat” principle, depletion of the mucus at one site may quickly be compensated and replaced by mucus from adjacent zones [15]. This might have resulted in a quick or immediate repair of the mucus layer and resumed protection of the skin against bacterial invasion.

The absence of skin ulcerations in the non-treated zones might indicate that an intact skin indeed provides an adequate barrier against *V. tapetis* infection and/or colonization. Based on these results, *V. tapetis* is considered to be a facultative pathogen as already previously reported [28, 33, 34].

The asymmetry of flatfish has since long been a fascinating topic [10, 21]. Although one might expect morphological or even functional differences between both sides, merely a handful of studies investigated this phenomenon. Faílde et al. [35] showed that, besides a difference in pigmentation, a higher number of mucus-producing goblet cells with an occasional observation of clusters of such cells were found on the pigmented compared to the non-pigmented side in turbot (*Psetta maxima*). In the current study, more, severe and larger ulcerations developed on the pigmented side indicating a higher susceptibility. In contrast, Vethaak [22] reported higher incidences of skin ulcerations on the non-pigmented side in wild-caught flounder (*Platichthys flesus*). Wiklund and Bylund [19] reported a sex-related distribution of ulcerations on pigmented and non-pigmented side of wild-caught flounder. The reason(s) for these apparently divergent findings hitherto remain(s) obscure.

It should be kept in mind that in general, skin ulcerations in fish are considered to have a multifactorial cause [20]. Therefore, it may be assumed that other factors, such as host-related characteristics (age, gender, immunity), can influence the development of skin ulcerations. Gender is frequently pointed out in literature as a predisposing factor for skin ulcerations [19, 20, 36]. The current study does not allow drawing conclusions on a possible difference in susceptibility between males and females due to the low amount of male fish.

Questions may rightfully arise on the impact of these ulcerations on the health and survival of the fish. In literature, effects such as loss of appetite [37], loss of reflexes [29], osmotic imbalance [20] and changing swimming behavior [37] are mentioned. Based on the information gathered in this study, one may speculate that the infection with *V. tapetis* and subsequent development of skin ulcerations impacts the survival of the fish as a peak in mortality of challenged fish was observed at 4 dpi, and overall, the mortality in challenged fish (55.6%) was significantly higher compared to control fish (16.7%). Septicemia caused by *V. tapetis* was already reported in corkwing wrasse and Atlantic halibut [3, 4]. Nevertheless, in the study of Lopez et al. [5], *V. tapetis* was only isolated from ulcerations and not from internal organs of wedge sole. In our study, a monoculture of *V. tapetis* was isolated from the spleen or liver of three fish that had died. However, since only a small number of bacterial isolates could be gathered of the dead fish due to post-mortem decay, the results were inconclusive. Beside septicemia, the engendered osmotic imbalance might also have induced mortality. Indeed, the skin is an important organ for maintaining the osmotic balance, whereby damage, as observed in skin ulcerations might cause a shift from an osmotic balance to an imbalance, leading to death [38]. Damage covering as little as 10% of the body surface area was reported to cause high mortalities [11]. This reasoning together with the finding that the skin ulcerations in the fish that died were generally more severe than those in surviving fish, contribute to rectifying the hypothesis that the observed mortalities were due to osmotic imbalance. Blood analysis in future experiments might corroborate this reasoning [39, 40].

In the present study, wild-caught common dab was included contributing to the representativeness of the obtained results in the field. Nevertheless, working with wild-caught fish inevitably entails that no information on the history or characteristics of the fish is available which most likely will increase the inter-individual variability and enhance the possibility for unforeseen complications. The latter greatly applies to the presence of (non-)infectious diseases. In this study, eight fish were included in the experiment with pre-existing small ulcerations (*n* = 4) or multifocal bulging lesions (1–2 mm) (*n* = 4) (data not shown). Furthermore, five other fish developed similar small bulging lesions during the experimental period. These (pre-existing) lesions were assumed to have no effect on the results of this study. In addition, a mortality of 16.7% was noted in the control group for which no cause was established. The same observation was made when wild corkwing wrasse was kept in captivity with typically 1–5% daily mortality in the population starting 1 month after capture [28]. From fish both from the challenge and control group, *V. tapetis* was recovered before the trial was initiated (data not shown) and following necropsy during the experiment. These findings again suggest a facultative pathogenic nature of *V. tapetis*. This again rectifies the findings in the present study that prior skin damage acts as a major contributing factor to the development of skin ulcerations.

In conclusion, *Vibrio tapetis* is able to cause skin ulcerations although a breach of the skin barrier seems to be a major contributing factor to the development of skin ulcerations. The pinpointed infection model using bath immersion may be used in the future to study the role of various anthropogenic or environmental factors on the development of skin ulcerations.

## Additional files

**Additional file 1.** **Schematic overview of the experimental design.** On both pigmented and non-pigmented sides, three distinct treatment zones were defined; a mechanical, chemical and non-treatment zone. The sequence of all treatments was altered on each fish, resulting in six subgroups of each ten fish.

**Additional file 2.** **Scoring of the different parameters that are part of the “Gross Ulceration Score” (GUS).** The GUS is the sum of the score of all the parameters indicated below and represents the severity of an ulceration.

**Additional file 3.** **Daily mortality in control (black) and challenge group (grey) during the experimental period (days post-inoculation, dpi).** In the challenge group (55.6%), more individuals died during the experimental period compared to the control group (16.7%). Note the high peak in mortality at 4 dpi in the challenge group.

## Data Availability

The datasets used and/or analyzed during the current study are available from the corresponding author on reasonable request.

## Abbreviations

BNS: Belgian part of the North Sea
P: pigmented side of the fish
NP: non-pigmented side of the fish
RV: research vessel
TSA: tryptic soy agar
TSB: tryptic soy broth
MS-222: tricaine methanosulfonate
MT: mechanical treatment
CT: chemical treatment
NT: non-treatment
CFU: colony forming units
dpi: days post-inoculation
GUS: gross ulceration score
TA: total affected area
UA: ulcerative area
L: standard length
W_B_: weight at the beginning of the experimental period
W_E_: weight at the end of the experimental period
K_B_: Fulton condition factor at the beginning of the experimental period
K_E_: Fulton condition factor at the end of the experimental period
TCBS: thiosulfate citrate bile salt sucrose agar
H&E: hematoxylin and eosin
D: fish that died during the experimental period
S: fish that survived until 21 dpi
