# Supplementary material for: Scrutinizing the triad of Vibrio tapetis, the skin barrier and pigmentation as determining factors in the development of skin ulcerations in wild common dab (Limanda limanda)
Source: Vet Res. 2019 Jun 3;50:41. doi: 10.1186/s13567-019-0659-6 (PMC6547549; doi:10.1186/s13567-019-0659-6)
Supplement: Supplementary file 1 — Additional file 1. Schematic overview of the experimental design. On both pigmented and non-pigmented sides, three distinct treatment zones were defined; a mechanical, chemical and non-treatment zone. The sequence of all treatments was altered on each fish, resulting in six subgroups of each ten fish. [file 13567_2019_659_MOESM1_ESM.pdf]

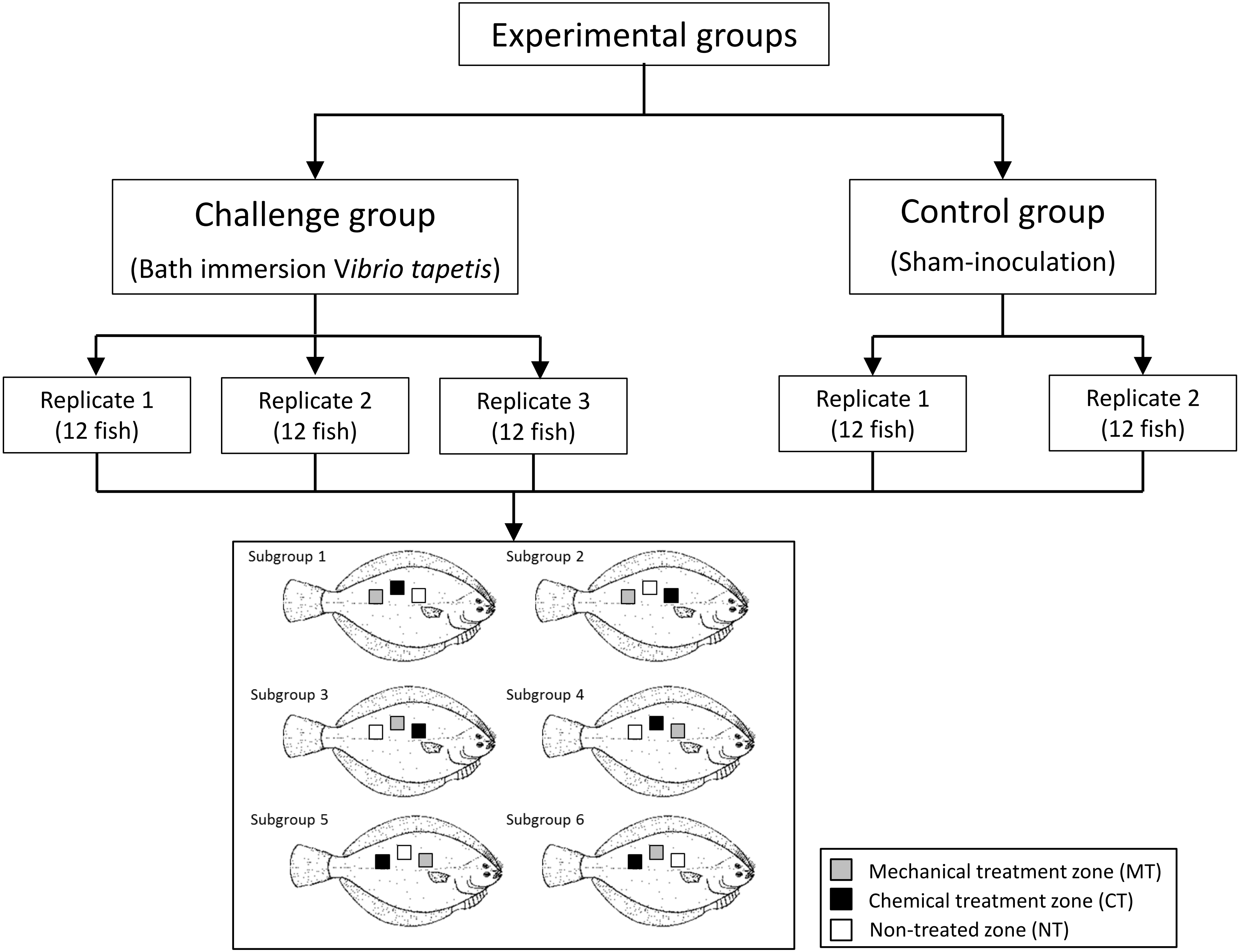
Experimental groups
Challenge group
(Bath immersion *Vibrio tapetis*)
Control group
(Sham-inoculation)
Replicate 1
(12 fish)
Replicate 2
(12 fish)
Replicate 3
(12 fish)
Replicate 1
(12 fish)
Replicate 2
(12 fish)
Subgroup 1
Subgroup 2
Subgroup 3
Subgroup 4
Subgroup 5
Subgroup 6
Mechanical treatment zone (MT)
Chemical treatment zone (CT)
Non-treated zone (NT)
