# Supplementary figures and images for: Scrutinizing the triad of Vibrio tapetis, the skin barrier and pigmentation as determining factors in the development of skin ulcerations in wild common dab (Limanda limanda)
Source: Vet Res. 2019 Jun 3;50:41. doi: 10.1186/s13567-019-0659-6 (PMC6547549; doi:10.1186/s13567-019-0659-6)

Mortality  
(%)

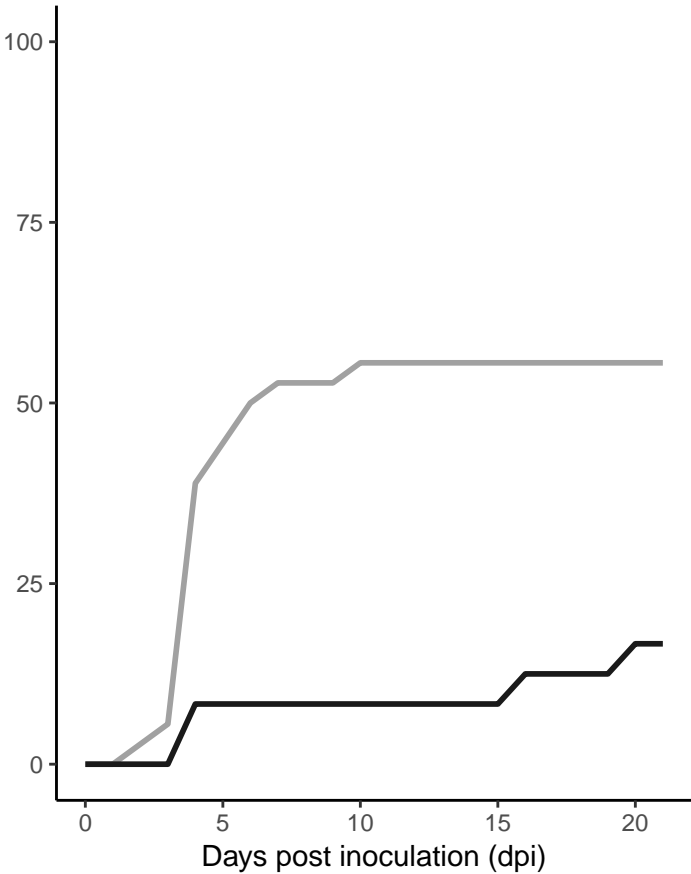

Supplement: Supplementary file 3 — Additional file 3. Daily mortality in control (black) and challenge group (grey) during the experimental period (days post-inoculation, dpi). In the challenge group (55.6%), more individuals died during the experimental period compared to the control group (16.7%). Note the high peak in mortality at 4 dpi in the challenge group. [file 13567_2019_659_MOESM3_ESM.pdf]
